# A Novel TLR4-Mediated Signaling Pathway Leading to IL-6 Responses in Human Bladder Epithelial Cells

**DOI:** 10.1371/journal.ppat.0030060

**Published:** 2007-04-27

**Authors:** Jeongmin Song, Matthew J Duncan, Guojie Li, Cheryl Chan, Richard Grady, Ann Stapleton, Soman N Abraham

**Affiliations:** 1 Department of Molecular Genetics and Microbiology, Duke University Medical Center, Durham, North Carolina, United States of America; 2 Department of Pathology, Duke University Medical Center, Durham, North Carolina, United States of America; 3 Program in Cell and Molecular Biology, Duke University Medical Center, Durham, North Carolina, United States of America; 4 Children's Hospital and Regional Medical Center, Seattle, Washington, United States of America; 5 Department of Microbiology, University of Washington, Seattle, Washington, United States of America; 6 Department of Immunology, Duke University Medical Center, Durham, North Carolina, United States of America; Washington University School of Medicine, United States of America

## Abstract

The vigorous cytokine response of immune cells to Gram-negative bacteria is primarily mediated by a recognition molecule, Toll-like receptor 4 (TLR4), which recognizes lipopolysaccharide (LPS) and initiates a series of intracellular NF-κB–associated signaling events. Recently, bladder epithelial cells (BECs) were reported to express TLR4 and to evoke a vigorous cytokine response upon exposure to LPS. We examined intracellular signaling events in human BECs leading to the production of IL-6, a major urinary cytokine, following activation by Escherichia coli and isolated LPS. We observed that in addition to the classical NF-κB–associated pathway, TLR4 triggers a distinct and more rapid signaling response involving, sequentially, Ca^2+^, adenylyl cyclase 3–generated cAMP, and a transcriptional factor, cAMP response element–binding protein. This capacity of BECs to mobilize secondary messengers and evoke a more rapid IL-6 response might be critical in their role as first responders to microbial challenge in the urinary tract.

## Author Summary

In spite of frequent cross contamination by bacteria from the gut, urinary tract infections are relatively infrequent. Although much of the credit goes to cells lining the urinary tract, such as bladder cells, how this is achieved remains unclear. Human bladder cells display, on their surfaces, special molecules called Toll-like receptors, which sense the presence of bacteria and trigger the cells to release a variety of chemicals called cytokines. Cytokines contribute to the recruitment of phagocytic cells from the blood to the site of infection to clear bacteria. In this paper, we reveal that the Toll-like receptor–initiated intracellular signals leading to the production of cytokines by bladder cells involve the same pathway seen in other cells, as well as an additional and more rapid signaling pathway. Rapid production of cytokines by bladder cells will facilitate early clearance of bacteria. Additionally, possession of multiple signaling pathways by bladder cells for producing cytokines is advantageous because bacteria that infect the urinary tract have the capability to suppress certain signaling events that lead to cytokine production by bladder cells.

## Introduction

The innate immune system is the first line of defense against infection and is thought to primarily be mediated by phagocytic immune cells such as macrophages and dendritic cells. These cells recognize microorganisms via a limited number of germline-encoded pattern recognition receptors (PRRs) that recognize microbial components known as pathogen-associated molecular patterns, which are essential for the survival of the microorganism and, therefore, difficult for the microorganism to alter [1]. Several classes of PRRs, including Toll-like receptors (TLRs) and cytoplasmic receptors, recognize distinct microbial components and directly activate immune cells, triggering intracellular signaling cascades that rapidly induce the expression of a variety of inflammatory cytokines that initiate a variety of overlapping immune responses. One of the best known PRRs is TLR4, which recognizes the major Gram-negative bacterial surface component lipopolysaccharide (LPS) [1]. Studies on TLR4 signaling in monocytes, macrophages, and dendritic cells have revealed that engagement of TLR4 by LPS triggers a signaling cascade involving several intracytoplasmic and nuclear transcriptional factors. TLR4 activation first engages a set of adaptor family members that link TLR4 to the serine/threonine kinases. These kinases mediate phosphorylation and ubiquitination of various substrates, eventually resulting in the activation of the transcriptional factor NF-κB, which regulates the expression of several immunomodulatory cytokines [2].

The urinary tract is extremely intractable to infection by most pathogens. This is attributable to a large extent on the multifaceted innate immune defenses of the bladder and, in particular, bladder epithelial cells (BECs). These cells selectively exfoliate upon bacterial colonization and undergo re-epithelialization as a mechanism to reduce bacterial load in the bladder. They are also a major source of proinflammatory cytokines and chemokines in the urinary tract following bacterial infection [3,4]. These BEC-derived mediators are responsible for the vigorous neutrophil response, which is responsible for early clearance of infecting bacteria [5]. A prominent mediator released by BECs is IL-6 and it is, by far, the single most prominent cytokine detected in the urine of infected patients [6]. IL-6 is known to mobilize and amplify both local as well as systemic innate immune defenses against infection [7]. The production of some of the earliest indicators of inflammation in the body such as the acute phase proteins has been directly related to production of this cytokine [7].

Considering the large number of pathogens capable of infecting the urinary tract, it is remarkable that uropathogenic Escherichia coli (UPEC) account for over 85% of urinary tract infections in patients without underlying predisposing factors [8]. The singular success of UPEC in the urinary tract has been attributed to bacterial surface expression of filamentous fimbrial appendages, called type 1 fimbriae [9]. These structures promote avid bacterial binding to uroplakin 1a molecules on the surface of BECs, triggering bacterial invasion of these cells [10,11]. In their intracellular location, UPEC avoid elimination by the flushing actions of urine [12]. Recent studies have suggested additional traits on UPEC that account for their success as uropathogens. These include their capacity to block apoptosis and exfoliation of infected BECs [13] as well as inhibit the ability of BECs to mount a cytokine responses [14]. Although several genes on UPEC have been implicated in inhibiting cytokine production, the underlying mechanism remains elusive, a problem exacerbated, at least partly, by the fact that most of our current understanding of TLR4 signaling is based almost exclusively on cells of hematopoietic origin [1]. Here, we sought to better define LPS/TLR4 signaling pathway in BECs. We were especially interested in defining the role, if any, of two second messengers, Ca^2+^ and cyclic adenosine monophosphate (cAMP), since these low molecular weight diffusible molecules have been globally implicated in cellular signaling events, including cytokine responses. We investigated the IL-6 responses of human BECs to E. coli and to isolated LPS. Our studies demonstrated that the IL-6 response triggered by TLR4 in BECs involves not only the classical NF-κB–associated pathway, but also a distinct pathway involving Ca^2+^, cAMP, and cAMP response element–binding protein (CREB). Interestingly, the latter pathway resulted in a significant IL-6 response, which is evident at least 3 h before the NF-κB–associated pathway.

## Materials and Methods

### Bacteria and BECs

A K-12 laboratory *E. coli* strain ORN103(pSH2) and a UPEC type 1 fimbriated and non-hemolytic strain CI5 were utilized in this study [15–17]. Bacteria and the human BEC line 5637 (ATCC HTB-9) and primary BECs were cultured as described previously [11,18]. Human airway epithelial cells (16-HBE) were cultured in DMEM plus 4 mM glutamine and 10% FBS, and the human monocytic cell line (Mono Mac 6) was cultured as described previously [19].

### IL-6 Measurements

IL-6 secretion was tested by using the human IL-6 ELISA kit (R&D Systems, http://www.rndsystems.com) according to the manufacturer's protocol. Cell viability was not affected by any of the pharmacological agents employed, as assessed by trypan blue exclusion assays.

### Ratiometric Imaging

Ratiometric Ca^2+^ imaging was performed as described previously [20]. The Fura-2 calcium imaging calibration kit (Molecular Probes, http://probes.invitrogen.com) was used to calibrate fluorimetric analyses to quantify intracellular calcium concentrations.

### Measurement of Intracellular cAMP Levels

Intracellular concentrations of cAMP were determined using a cAMP enzyme immunoassay kit (Sigma, http://www.sigmaaldrich.com) according to the manufacturer's instructions.

### RNA Isolation and RT-PCR

Total cellular RNA was isolated using an RNeasy purification system (Qiagen, http://www.qiagen.com). Two micrograms of total RNA was reverse transcribed and amplified with gene-specific primers using the RT-PCR System kit (Bio-Rad, http://www.bio-rad.com). The primer sequences for the genes and expected product sizes are summarized in Table S1. We confirmed that the adenylyl cyclase (AC) isotype-specific primers were functional by undertaking reverse transcription (RT)–PCR on total RNA from HEK cells (a positive control cell, where all ACs except AC-4 and AC-8 were expressed) [21].

### Creation of Knockdowns Using RNA Interference

Detailed methods and target sequences, including GenBank accession numbers for the genes mentioned in this study, are described in Text S1.

### Western Blot Analysis

Detailed methods and materials are described in Text S1.

### Detection of NF-κB Nuclear Translocation

Nuclear extraction kit (Chemicon, http://www.chemicon.com) was used for performing a nuclear extraction, and the active form of NF-κB contained in the nuclear extract was detected using an NF-κB p65 Transcription Factor assay system (Chemicon).

### CREB Binding Assay

We employed the Noshift transcription factor assay system (Novagen, http://www.emdbiosciences.com/html/NVG/home.html) to assay binding of CREB to CRE oligonucleotides. BECs were cultured and exposed for 1 h to E. coli ORN103(pSH2), and nuclear extracts were collected following the vendor's recommendation (Novagen). To detect the binding of CREB to the CRE site of the IL-6 promoter, CRE oligonucleotides from IL-6 promoter region were synthesized and end-labeled by biotinylation. The CRE oligonucleotide sequences utilized were the same as those used previously [22].

## Results

### IL-6 Response of BECs to Type 1 Fimbriated E. coli Is Largely Elicited by LPS and Involves TLR4

**Figure 1:**
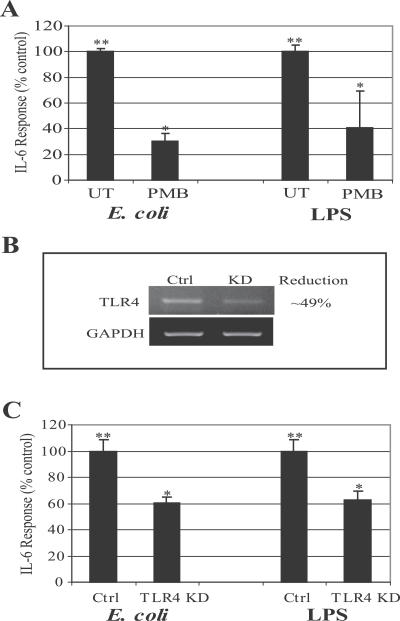
IL-6 Response of BECs to Type 1 Fimbriated E. coli Is Largely Elicited by LPS and Involves TLR4 (A) IL-6 secretion by BECs in response to E. coli (100 multiplicity of infection) or purified LPS (100 μg/ml). When specified, E. coli and purified LPS were pretreated with 1 μg/ml PMB for 30 min. **, *p* < 0.001 relative to values of untreated (UT) BECs; *, *p* < 0.03 relative to E. coli or LPS-treated BECs. (B) RT-PCR of control-transfected BECs (Ctrl) and TLR4 KD BECs. Glyseraldehyde-3-phosphate dehydrogenase (GAPDH) was employed as a loading control. (C) IL-6 secretion of control-transfected BECs (Ctrl) and TLR4 KD BECs after E. coli and LPS stimulation. **, *p* < 0.05 relative to UT control; *, *p* < 0.05 relative to E. coli-treated control or LPS control.

Although there are several data implicating type 1 fimbriae and its adhesive subunit, FimH, as the determinant largely responsible on UPEC for triggering endocytic responses from BECs [10,11], a recent study has reported LPS as the primary determinant on UPEC responsible for evoking the cytokine response from BECs [23]. We initiated our studies by examining the role of LPS in mediating the IL-6 response of BECs following exposure to E. coli. This was undertaken by comparing the IL-6 response of the human BEC line 5637 to E. coli in the presence and absence of polymyxin B (PMB), which binds to the lipid A portion of LPS and blocks its recognition by host cells [23]. The E. coli strain we selected for our studies was a well-characterized laboratory strain of E. coli ORN103(pSH2) expressing recombinant type 1 fimbriae, including the adhesive subunit, FimH. We employed this laboratory strain rather than a UPEC strain because UPEC strains express multiple genes capable of suppressing cytokine responses in BECs [14]. We observed a strong IL-6 response from BECs following exposure to the laboratory E. coli that was significantly reduced following pretreatment of the bacteria with PMB (Figure 1A). For comparative purposes, shown in Figure 1A is the PMB-mediated inhibition of the IL-6 responses of BECs to soluble E. coli LPS. Due to the possibility of lipoprotein contamination of LPS prepared by trichloroacetic acid (TCA) or phenol-chloroform-petroleum ether (PCP) extraction, LPS ultra purified by ion exchange chromatography and verified to contain <1% protein was used in this study (Sigma; E. coli 055:B5 LPS). To confirm that the LPS on E. coli was the primary determinant responsible for activating BECs, we sought to show that the activation of BECs involved TLR4, the signaling receptor for LPS. Using RNA interference (RNAi) techniques, we generated BECs where expression of TLR4 was appreciably knocked down. Densitometric quantification of message levels in the knockdown (KD) BECs revealed that the expression of TLR4 was reduced by 49% (Figure 1B). Shown in Figure 1C is the IL-6 response of control (transfected with control vector) BECs and of the KD BECs to E. coli and LPS. Compared to control BECs, significant reduction in the IL-6 response to both E. coli and LPS was observed with the KD cells (Figure 1C). For the most part, the reduction in the IL-6 response paralleled the degree of KD of TLR4 in the BECs (Figure 1B–1C). Taken together, these data confirm that LPS is the primary determinant on E. coli responsible for triggering the IL-6 response, and the intracellular signaling triggered by LPS involves TLR4 on BECs.

### IL-6 Response of BECs to E. coli Is Preceded by an Increase in Intracellular Ca^2+^

**Figure 2:**
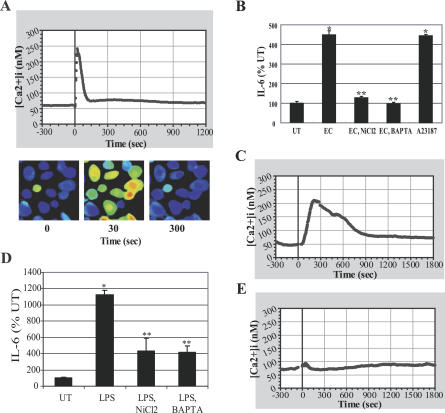
IL-6 Response of BECs to *E. coli* Is Preceded by an Increase in [Ca^2+^]_i_ (A) [Ca^2+^]_i_ tracing of BECs before and after E. coli exposure. E. coli was added at time 0. (B) BEC IL-6 responses after E. coli exposure in the absence or presence of NiCl_2_ (2 mM) or BAPTA-AM (5 μM), or after calcium ionophore A23187 (1 μM) treatment without bacterial exposure. *, *p* < 0.001 relative to untreated (UT) BECs; **, *p* < 0.001 relative to E. coli (EC)–treated BECs. (C–E) BEC [Ca^2+^]_i_ tracing before and after purified LPS treatment (C) or PMB pretreated LPS treatment (E). (D) BEC IL-6 responses following exposure to LPS in the absence or presence of NiCl_2_ or BAPTA-AM. *, *p* < 0.001 relative to UT BECs; **, *p* < 0.01 relative to LPS-treated BECs.

Since intracellular Ca^2+^ ([Ca^2+^]_i_) has been implicated in important cellular processes, including IL-6 secretion [20,24], we examined the involvement, if any, of this second messenger in the IL-6 response of BECs to E. coli. We investigated whether exposure of BECs to E. coli induced an increase in [Ca^2+^]_i_ by performing ratiometric imaging on Fura-2/AM-loaded 5637 BECs. A unique pattern of Ca^2+^ influx into exposed BECs to E. coli was observed (Figure 2A). BEC [Ca^2+^]_i_ was constant before bacterial exposure and increased rapidly, within 1 min, after E. coli exposure, returning to baseline levels within 5 min (Figure 2A). To determine whether the E. coli–induced increase of [Ca^2+^]_i_ was essential for the BEC IL-6 response to bacterial exposure, we examined IL-6 secretion by BECs following bacterial exposure with or without pretreatment with NiCl_2_, a general Ca^2+^ channel inhibitor [25], or BAPTA-AM, a [Ca^2+^]_i_ chelator [26] (Figure 2B). Whereas BEC IL-6 secretion was readily induced after exposure to *E. coli,* pretreatment of BECs with NiCl_2_ or BAPTA-AM before bacterial exposure completely abolished IL-6 secretion by exposed BECs to E. coli. In addition, a general inducer of calcium influx, ionophore A23187, was able to directly induce BEC IL-6 production in the absence of *E. coli,* demonstrating the importance of [Ca^2+^]_i_ increases in initiating this response.

We also investigated whether purified E. coli LPS was capable of inducing an increase in [Ca^2+^]_i_ in BECs. LPS was seen to induce a similar, but delayed, increase in [Ca^2+^]_i_ compared to that caused by E. coli (Figure 2A and 2C). The LPS-induced [Ca^2+^]_i_ peak occurred ~5 min after the addition of 100 μg/ml LPS. Disrupting the LPS-induced [Ca^2+^]_i_ increase with NiCl_2_ or BAPTA-AM pretreatment before LPS exposure greatly reduced IL-6 production by BECs (Figure 2D). Pretreatment of LPS with PMB almost completely abrogated the [Ca^2+^]_i_ response of BEC (Figure 2E). Taken together, these observations provide strong evidence indicating that the IL-6 response of BECs to E. coli involves a sharp increase in [Ca^2+^]_i_ levels. Although LPS appears to be the primary bacterial component responsible for elevation of [Ca^2+^]_i_, bacteria-associated LPS evoked a faster [Ca^2+^]_i_ response in BECs compared to that of soluble LPS.

### IL-6 Response of BECs to E. coli Is Associated with a Significant Increase in Intracellular cAMP Levels

**Figure 3:**
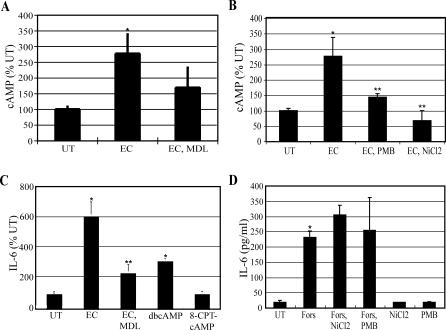
IL-6 Response of BECs to E. coli Is Associated with a Significant Increase in Intracellular cAMP Levels (A and B) BEC cAMP production before and after E. coli exposure. When specified, BECs were pretreated with MDL-12,330A (MDL) (0.4 mM) or NiCl_2_ for 30 min, or E. coli was pretreated with PMB for 30 min. *p < 0.03 relative to untreated (UT) BECs; **p < 0.03 relative to E. coli (EC)–treated BECs. (C) IL-6 secretion by BECs was measured 6 h after exposure to *E. coli,* in the absence (EC) or presence of MDL-12,330A (EC, MDL), or after 6 h of treatment with 1 mM dibutyryl cAMP (dbcAMP), or 1 mM 8-(4-chloro-phenylthio)-2′-O-methyladenosine-3′-5′-cyclic monophosphate (8-CPT-cAMP) without bacterial exposure. *, *p* < 0.01 relative to UT; **, *p* < 0.02 relative to EC. (D) IL-6 secretion by BECs incubated for 6 h in the absence (UT) or presence of the AC-activator forskolin (Fors) (50 μM) with or without NiCl_2_ or PMB, or incubated with NiCl_2_ or PMB in the absence of forskolin. *, *p* < 0.01 relative to UT BECs.

Intracellular cAMP is an important second messenger in several signaling pathways, including IL-6 response [27–29]. Exposing BECs to E. coli for 1 h demonstrated a 2.7-fold increase in intracellular cAMP, which was blocked by inhibiting AC activity with the compound MDL-12,330A (MDL) (Figure 3A). The increase in intracellular cAMP following bacterial exposure was dependent on both bacteria-associated LPS and an increase in [Ca^2+^]_i_ as shown, respectively, by pretreating the bacteria with PMB or pretreating the BECs with NiCl_2_ (Figure 3B). This E. coli–induced [Ca^2+^]_i_-dependent cAMP production was found to be an important step in the cytokine response of BECs to bacterial exposure, since inhibition of ACs with MDL reduced BEC IL-6 expression by ~75% (Figure 3C). In addition, a membrane-permeable cAMP analog, dibutyryl cAMP, induced a greater than 3-fold increase in BEC IL-6 production in the absence of bacterial exposure, demonstrating the importance of intracellular cAMP in inducing BEC IL-6 production. However, a membrane-permeable cAMP analog (8-CPT-cAMP) that does not activate the classical cAMP-target protein, protein kinase A (PKA), but only activates the recently discovered cAMP-target protein Epac (exchange protein activated by cAMP) [30], did not induce the production of BEC IL-6, indicating that PKA is involved in the downstream induction of IL-6 production by exposed BECs to E. coli (Figure 3C). Forskolin activates ACs, the enzymes that produce intracellular cAMP, by a direct mechanism [31], which should bypass the need for an increase in [Ca^2+^]_i_ that is observed with E. coli–induced intracellular cAMP production. As shown in Figure 3D, direct activation of AC by forskolin led to a dramatic production of IL-6 that was not inhibited by NiCl_2_, indicating that the increase in [Ca^2+^]_i_ that occurred after E. coli exposure preceded the production of intracellular cAMP. Neither NiCl_2_ nor PMB treatment affected forskolin-induced IL-6 production, demonstrating that these agents did not have a detrimental effect on protein synthesis in general (Figure 3D). Thus, the IL-6 response to E. coli evoked by BECs involves another secondary messenger, cAMP, which acts downstream of the Ca^2+^ response.

### AC-3 Is Responsible for Mediating E. coli–Induced cAMP in BECs

**Figure 4:**
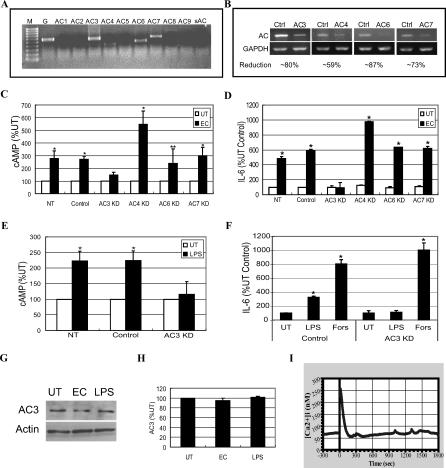
AC-3 Is responsible for Mediating E. coli–Induced cAMP Production in BECs (A) RT-PCR of BECs using primers specific for the ten known mammalian AC isoforms. Only AC-3, AC-4, AC-6, and AC-7 mRNA was expressed. GAPDH-specific RT-PCR (lane G) was used as a loading control. M, marker; sAC, soluble AC. (B) RT-PCR of control-transfected BECs (Ctrl) and AC-3, AC-4, AC-6, or AC-7 KD BECs. GAPDH-specific RT-PCR was used as a loading control. (C and D) Intracellular cAMP production (C) and IL-6 secretion (D) by non-transfected BECs (NT), control-transfected BECs (Control), or AC-3, AC-4, AC-6, or AC-7 KD BECs left untreated (UT) or treated with E. coli (EC). *, *p* < 0.005 and **, *p* < 0.02 relative to respective UT values. (E and F) Intracellular cAMP production (E) and IL-6 secretion (F) by non-transfected BECs (NT), control-transfected BECs (Control), or AC-3 KD BECs were measured in the absence (UT) or presence of E. coli LPS, or presence of forskolin (Fors) without LPS. *, *p* < 0.003 relative to respective UT values. (G) BEC AC-3–specific Western blot before (UT) and after (EC) E. coli exposure for 1 h, or exposure to E. coli LPS for 6 h. An actin-specific Western blot was used as a loading control. (H) Densitometric analysis of AC-3–specific Western blots, using ImageJ software. (I) [Ca^2+^]_i_ tracing in AC-3 KD BECs before and after E. coli exposure. E. coli was added at time 0.

Because there are currently ten known isoforms of mammalian ACs [32] it was of interest to determine which AC was responsible for the E. coli–induced increase of intracellular cAMP in BECs. First, we determined which AC isoforms were actually expressed in BECs. RT-PCR was performed on total cellular RNA, using primers specific for each known AC isoform and only mRNA for AC isoforms AC-3, AC-4, AC-6, and AC-7 was detectable in BECs (Figure 4A). We confirmed that the other AC isotype-specific primers used were functional by undertaking RT-PCR on total RNA from human embryonic kidney (HEK) cells, positive control cells, where all ACs except AC-4 and AC-8 were expressed [21] (unpublished data). RNAi was utilized to minimize the expression of each AC, which was verified by AC isotype-specific RT-PCR (Figure 4B). Following E. coli exposure, intracellular cAMP, as well as IL-6 secretion, rose significantly in all of the KDs, except for the KD of AC-3, indicating that AC-3 is the BEC AC isoform linked to the IL-6 response following E. coli exposure (Figure 4C and 4D). It is noteworthy that of the four AC isoforms expressed by BECs, only AC-3 is known to be activated by increases in [Ca^2+^]_i_ [33,34]. The KD of AC-3 also abrogated the production of intracellular cAMP (Figure 4E) and expression of IL-6 (Figure 4F) following exposure of BECs to purified LPS. Interestingly, in the AC-3 KD BECs, forskolin-induced IL-6 expression was largely unaffected (Figure 4F). The appreciable IL-6 response to forskolin suggested that a general increase in intracellular cAMP was sufficient to signal IL-6 secretion in BECs. The absence of any reduction in the IL-6 response to forskolin in AC-3 KD BECs is attributable to the presence of other isoforms of ACs in these cells, which were directly activated by forskolin. Remarkably, when AC-3–specific Western blotting was performed on BECs before and after bacterial exposure or exposure to purified LPS, no discernible increase in the expression of AC-3 protein was observed, indicating that an increase in activity, rather than expression, of AC-3 occurred following infection (Figure 4G and 4H). Finally, when we examined for increase in [Ca^2+^]_i_ in the AC-3 KD BECs following exposure to *E. coli,* we found that it was comparable to that seen in wild-type (WT) BECs (Figure 4I), which is consistent with the idea that the rise in [Ca^2+^]_i_ preceded any rise in intracellular cAMP.

### cAMP-Mediated Phosphorylation of the CREB

**Figure 5:**
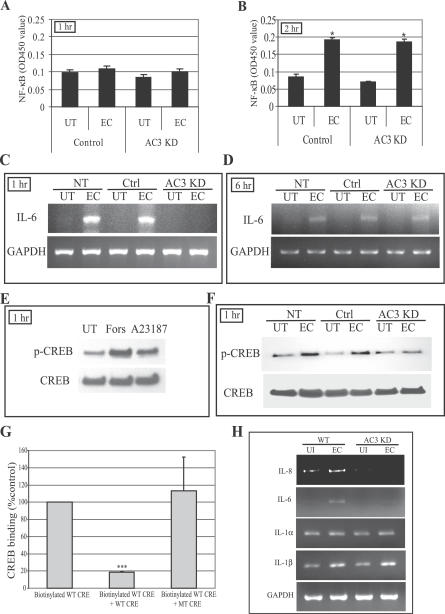
cAMP-Mediated Phosphorylation of CREB and Binding of CREB to CRE Oligonucleotides (A and B) NF-κB nuclear translocation in control-transfected BECs and AC-3 KD BECs before and after E. coli (EC) exposure for 1 h (A) or 2 h (B). UT, untreated. (C and D) IL-6 message levels in non-transfected (NT), control-transfected (Ctrl), and AC-3 KD BECs before and after *E. coli* exposure for 1 h (C) or 6 h (D) as measured by RT-PCR. (E) Western blot of CREB phosphorylation levels (p-CREB) in UT BECs, and forskolin (Fors)– or calcium ionophore A23187 (A23187)–treated BECs. The treatment was for 1 h. (F) Western blot showing CREB phosphorylation of NT, Ctrl, and AC-3 KD BECs before and after 1 h *E. coli* exposure. (G) CREB binding to CRE site of the IL-6 promoter. Nuclear extracts of BECs exposed for 1 h to E. coli ORN103(pSH2) were incubated with biotinylated WT CRE oligonucleotides in the absence (Biotinylated WT CRE) or presence of specific (Biotinylated WT CRE + WT CRE) or non-specific (Biotinylated WT CRE + MT CRE) oligonucleotide competitors. ***, *p* < 0.0001. (H) Expression analysis of mRNA levels of various genes with CRE sites in their promoter. WT and AC-3 KD BECs were incubated for 1 h with E. coli ORN103(pSH2), and then total RNA was collected from untreated (UI) and bacteria-treated BECs (EC) and subjected to RT-PCR. GAPDH was used as a loading control.

Next, we sought to connect the Ca^2+^- and cAMP-dependent signaling events described in this study to the classical NF-κB–associated signaling pathway mediated by TLR4. To link cAMP to the classical pathway, we examined its effects on the translocation of the transcriptional factor NF-κB from the cytoplasm to the nucleus following bacterial exposure. Remarkably, when we examined control-transfected BECs and AC-3 KD BECs 1 h following exposure to E. coli (which corresponds to the time we observed significant secondary messenger responses) for nuclear translocation, we found little or no translocation of NF-κB in either cell type (Figure 5A). However, when we increased the incubation time to 2 h following exposure to *E. coli,* we detected a marked increase in translocation of NF-κB in control-transfected BECs, and an identical increase was also seen in AC-3 KD BECs (Figure 5B). This finding revealed that (i) the cAMP responses in BECs preceded the nuclear translocation of NF-κB by significant amounts of time, and (ii) these cAMP responses did not appear to impact the NF-κB–associated signaling pathway. These observations raised the intriguing possibility that secondary messengers such as cAMP may be acting via an independent pathway. To see whether the regulatory effect of cAMP on the IL-6 response was at the transcriptional level, we compared IL-6 mRNA levels in WT BECs, control-transfected BECs, and AC-3 KD BECs before and 1 h after exposure to E. coli. We observed a marked increase in IL-6 mRNA in WT BECs and control-transfected BECs but not in AC-3 KD BECs (Figure 5C), indicating that the AC-3–mediated elevation in intracellular cAMP was regulating the IL-6 response at the transcriptional level. It is pertinent to also note the time frame of when these assays were undertaken. Here, mRNA for IL-6 was detected in control-transfected BECs as early as 1 h after exposure to E. coli (Figure 5C). Considering that nuclear translocation of NF-κB was detectable only after 2 h (Figure 5B), this cAMP-regulated pathway appears to be activated sooner than the classical pathway. Interestingly, when we examined AC-3 KD BECs for IL-6 mRNA 6 h after exposure to *E. coli,* we detected similar amounts of message in AC-3 KD BECs and control-transfected BECs (Figure 5D), indicating that the classical NF-κB–mediated pathway was still functional in AC-3 KD BECs. Thus, the IL-6 response in BECs appears to originate from two distinct pathways: the NF-κB–associated pathway and a separate but speedier pathway involving Ca^2+^ and cAMP. One mechanism through which cAMP may directly affect transcription of IL-6 is by promoting phosphorylation of CREB, which binds to CRE in the IL-6 promoter region [35]. An increase in intracellular cAMP levels activates PKA, whose catalytic subunits enter the nucleus and phosphorylates CREB [36]. Upon phosphorylation, CREB promotes the recruitment of various transcriptional co-activators that promote transcription of target genes with consensus sites for CREB, such as IL-6 [37,38]. When we examined for CREB phosphorylation in BECs exposed to forskolin and calcium ionophore A23187, two potent elevators of intracellular cAMP, we observed a marked increase in CREB phosphorylation on the Western blots (Figure 5E) consistent with the idea that CREB phosphorylation occurred following elevation of intracellular cAMP levels. To see if bacterial exposure also triggered phosphorylation of CREB, CREB protein from extracts of BECs before and after exposure to E. coli was probed for phosphorylation. Following E. coli exposure, we found an appreciable increase in phosphorylation of CREB in non-transfected BECs and control-transfected BECs, but not in AC-3 KD BECs (Figure 5F). To extend these findings, we investigated the binding of CREB to the CRE sites on the IL-6 promoter region. Nuclear extracts of BECs were obtained after 1 h incubation with E. coli ORN103(pSH2) and then incubated with the biotinylated oligonucleotides corresponding to CRE on the IL-6 promoter. Binding of CREB to CRE was assessed by a colorimetric assay. We found that CREB bound to CRE oligonucleotides but not to a scrambled oligonucleotide sequence of identical length (Figure 5G). Thus, cAMP appears to be modulating IL-6 responses through the binding of the phosphorylated transcriptional factor CREB to the CRE site on the IL-6 promoter region.

Since there are other inflammatory mediators such as IL-1α, IL-1β, and IL-8 with consensus CRE sites in their promoter region that have known to be activated during urinary tract infection [14,39,40], we examined whether production of any of these mediators was modulated by the cAMP/CREB pathway following exposure to E. coli. We compared message levels for IL-1α, IL-1β, and IL-8 in WT and AC-3 KD BECs after 1 h exposure to E. coli ORN103(pSH2). We found that mRNA levels for IL-8 but not IL-1α or IL-1β appeared to be regulated by the TLR4/cAMP/CREB pathway. Thus, in addition to IL-6, production of IL-8 by BECs appears to be under the regulation of the novel signaling pathway.

### The IL-6 Response to E. coli of BECs Is the Product of Two Separate Signaling Pathways

**Figure 6:**
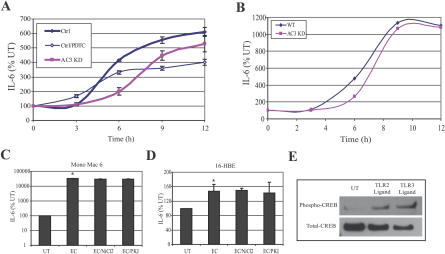
The IL-6 Response to E. coli of BECs Is the Product of Two Separate Signaling Pathways (A) IL-6 secretion response to E. coli ORN103(pSH2) by WT and AC-3 KD BECs in the absence or presence of pyrrolidine dithiocarbamate (PDTC). (B) IL-6 secretion response to UPEC CI5 by WT and AC-3 KD BECs. (C and D) IL-6 secretion responses by Mono Mac 6 cell line (C) and 16-HBE cell line (D). Cells were treated for 12 h with E. coli ORN103(pSH2) in the absence (EC) or presence of NiCl_2_ or PKA inhibitor (PKI). *, *p* < 0.03 relative to untreated (UT) value. Identical results were obtained after 6 h of treatment with E. coli ORN103(pSH2). (E) Increased CREB phosphorylation in BECs in response to TLR2 and TLR3 ligands. Western blotting for phospho-CREB and total-CREB in UT BECs or BECs treated with either a TLR2 ligand (lipoteichoic acid) or a TLR3 ligand (polyinosine-polycytidylic acid).

Based on the evaluation of transcriptional messages, the IL-6 response of BECs is mediated by two separate signaling pathways with different expression kinetics. To verify this observation, we compared the kinetics of IL-6 secretion in WT and AC-3 KD BECs following exposure to *E. coli.* We found that whereas appreciable IL-6 secretion (arbitrarily defined as 4-fold over unstimulated controls) was observed as early as 6 h in WT BECs, a comparable amount of IL-6 was only produced in AC-3 KD BECs after about 9 h (Figure 6A). By 12 h, however, the amounts of IL-6 secretion were not significantly different between both cell types, suggesting that the IL-6 responses of AC-3 KD BECs eventually caught up to that of the WT BECs (Figure 6A). A similar profile was obtained when we substituted the laboratory E. coli strain with a UPEC strain, CI5 (Figure 6B). To assess the relative contribution of the cAMP-mediated pathway to the IL-6 response of BECs, we examined E. coli–elicited IL-6 responses of WT BECs after selective inhibition of the NF-κB pathway with pyrrolidine dithiocarbamate [41]. This agent has been reported not to inhibit CREB activity [42]. We found that although the early kinetics of the IL-6 responses were not significantly different from untreated WT BECs, the amounts of IL-6 secreted, especially by the 12 h period of incubation, were significantly reduced. Thus, while the cAMP/CREB–mediated IL-6 response was an early one, the amounts of IL-6 generated by this pathway were significantly less than that produced by the classical NF-κB–associated pathway.

Additionally, we examined whether the IL-6 responses to E. coli mediated by other human cells involved the two second messengers, Ca^2+^ and cAMP. Monolayers of the human monocytic cell line Mono Mac 6, and the human bronchial epithelial cell line 16-HBE, were exposed to E. coli ORN103(pSH2) as before in the presence of inhibitors of either Ca^2+^ response or cAMP response, and IL-6 secretion was measured. We found that whereas both cell lines evoked appreciable IL-6 responses to *E. coli,* neither one of these responses were reduced by inhibitors of calcium (NiCl_2_) or cAMP (PKA inhibitor) signaling (Figure 6C and 6D). Thus, the two secondary messengers, Ca^2+^ and cAMP, appear to be important mediators of the IL-6 responses in BECs but not in other cell types.

Since BECs express other TLRs such as TLR2 and TLR3 [23,43], it was of interest to investigate whether known ligands for TLR2 and TLR3 also triggered the cAMP/CREB pathway. We assessed the phosphorylation levels of CREB before and 6 h after exposure to lipoteichoic acid (TLR2 ligand) or polyinosine-polycytidylic acid (TLR3 ligand) and found that both TLR ligands induced significant phosphorylation of CREB (Figure 6E). Thus, the CREB pathway appears to be activated by TLRs other than TLR4.

### Involvement of [Ca^2+^]_i_ and cAMP in the IL-6 Response of Primary Human BECs to UPEC

**Figure 7:**
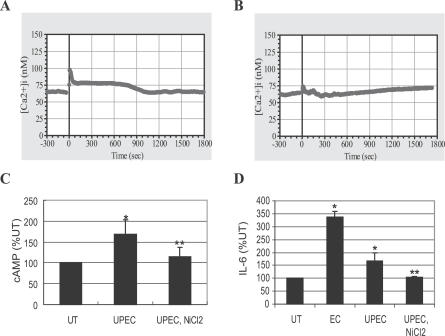
The IL-6 Response of Primary Human BECs to UPEC Is Linked to [Ca^2+^]_i_ and cAMP Increase (A) [Ca^2+^]_i_ tracing before and after exposure of primary human BECs to UPEC strain CI5. (B) [Ca^2+^]_i_ tracing of the primary human BECs pretreated with NiCl_2_ for 30 min. UPEC was added at time 0. (C and D) Intracellular cAMP production (C), or IL-6 secretion (D) by the primary human BECs left untreated (UT) or treated with E. coli CI5 (UPEC) in the absence or presence of NiCl_2_ (UPEC, NiCl_2_), or treated with E. coli ORN103 (EC). *, *p* < 0.03 relative to UT value. **, *p* < 0.03 relative to UPEC (CI5)–treated value.

Since the secondary messenger/CREB pathway was detectable only in immortalized human BECs, it was important to validate our observation of the existence of a cAMP/CREB pathway in primary human BECs. Therefore, we investigated whether freshly isolated and cultured human BECs would secrete IL-6 through a Ca^2+^- and cAMP-dependent mechanism when exposed to UPEC strain CI5 [16]. We cultured primary bladder cells obtained from fresh bladder biopsies as described previously [44]. These cells exhibited characteristics of primary BECs, including expression of uroplakin 1a, a marker of the asymmetrical unit membrane, the junctional complex protein ZO1, as well as cytokeratin, all of which are hallmarks of terminal differentiation in bladder umbrella cells (unpublished data). We observed that, after the stimulation with UPEC strain CI5, primary BECs exhibited elevation in [Ca^2+^]_i_ (Figure 7A). The intensity of the response was lower than that observed with the laboratory strain of E. coli (unpublished data). This is consistent with the fact that UPEC express multiple virulence factors, some of which exhibit disparate effects on [Ca^2+^]_i_ levels [20]. As demonstrated previously with 5637 BECs, this response was significantly abrogated following pretreatment with the general Ca^2+^ channel blocker, NiCl_2_ (Figure 7B). Significant elevation in intracellular cAMP levels and IL-6 secretion was observed in primary BECs following exposure to UPEC (Figure 7C and 7D). Both the increase in intracellular cAMP levels and IL-6 secretion were inhibitable by NiCl_2_, once again confirming the importance of [Ca^2+^]_i_ to the BEC IL-6 response (Figure 7C and 7D). Also shown in Figure 7D is the IL-6 response of these primary cells to the laboratory E. coli strain ORN103. Notice that it is markedly higher than the response to the UPEC strain. Taken together, these findings support the notion that although the IL-6 response of primary BECs to UPEC is dampened, it involves a Ca^2+^- and cAMP-dependent mechanism.

## Discussion

The bladder and the upper urinary tract are typically sterile, which is attributable, at least in part, to the highly efficient immune system monitoring these sites. One of the principal effectors of immune surveillance is the epithelial cell lining the urinary tract [3,45]. In addition to serving as a barrier against urine, BECs function as first responders, mobilizing multiple innate immune responses against microorganisms. Mediating microbial recognition on the surfaces of epithelial cells are PRRs, which recognize specific microbial products and activate intracellular signaling events leading to secretion of various inflammatory and immunoregulatory cytokines [43]. That epithelial cells possess PRRs such as TLR4 and contribute to immune surveillance has only recently been recognized [23,46]. For a long time it was assumed that PRRs were exclusively found on immune cells of hematopoietic origin and, therefore, most of our current information regarding PRR-mediated signal transduction is largely based on these cells [1,47]. Although there is no conclusive data suggesting cell-specific TLR4 signaling, there have been suggestions that LPSs evoke intracellular signaling reactions in Kupffer cells [48] and tracheal epithelial cells [49] that are absent in polymorphonuclear leukocytes [50]. Here, we report the existence of a distinct TLR4-mediated signaling pathway leading to IL-6 secretion that is present in BECs but absent in other human cell types.

This novel signaling pathway detected in BECs is independent of the classical pathway involving the transcriptional element NF-κB and contains two well-known secondary messengers, Ca^2+^ and cAMP, which mobilize a different transcriptional element, CREB. The existence of this pathway only became evident to us because of our focus on second messengers in TLR signaling and because our assays for IL-6 production were undertaken at earlier incubation periods than the more traditional 24–48 h incubation time points [7]. At the later incubation periods, the contribution of this novel pathway to the IL-6 response is superseded by the traditional NF-κB–mediated pathway. Evidence for the involvement of [Ca^2+^]_i_ in the early BEC IL-6 response comes from the finding that a flux in [Ca^2+^]_i_ was observed within a minute of exposure to *E. coli,* and inhibiting this flux with Ca^2+^ channel inhibitors or [Ca^2+^]_i_ chelators inhibited the IL-6 response (Figure 2B). Since a general inducer of calcium influx such as ionophore A23187 was able to induce IL-6 production from BECs even in the absence of *E. coli,* increase in [Ca^2+^]_i_ appears sufficient to trigger the IL-6 response from BECs (Figure 2B). Evidence for the role of cAMP in the IL-6 response comes from the finding that the IL-6 response to E. coli was closely associated with a 3-fold increase in intracellular levels of cAMP (Figure 3A). In addition, inhibition of cAMP-generating ACs significantly reduced the IL-6 response of BECs to E. coli (Figure 3C). As with the [Ca^2+^]_i_ flux, merely enhancing intracellular levels of cAMP with a membrane-permeable cAMP analog induced significant IL-6 release from BECs even in the absence of E. coli (Figure 3C). Thus, the two secondary messengers are sufficient, as well as necessary, for the early IL-6 secretion in BECs.

That Ca^2+^ response preceded the cAMP production following bacterial stimulation was deduced from the findings that (i) the earliest detectable increase in intracellular cAMP levels was observed 15 min following exposure to E. coli (unpublished data), whereas Ca^2+^ responses could be seen within a minute of bacterial exposure (Figure 2A), and (ii) the Ca^2+^ flux following exposure to E. coli remained largely unaffected in AC-3 KD BECs, while the cAMP response was abrogated (Figure 4I). Since enhancement of intracellular cAMP specifically required an increase in [Ca^2+^]_i_, we suspected that a Ca^2+^-inducible form of AC was responsible. BECs were found to express mRNA for AC-3, AC-4, AC-6, and AC-7, but only RNAi KD of AC-3, a Ca^2+^-inducible AC isoform, inhibited E. coli–induced intracellular cAMP production and subsequent IL-6 expression.

**Figure 8:**
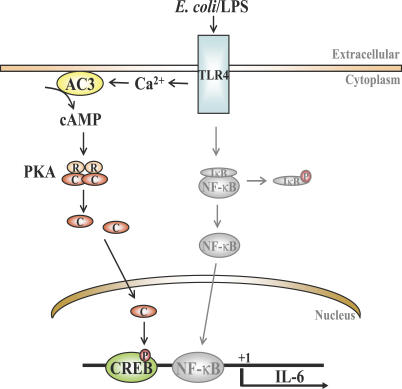
Proposed Model for TLR4 Signaling in BECs The proposed rapidly induced second messenger– and CREB-mediated pathway (dark line) as well as the classical NF-κB (grey line) are shown. Both pathways are triggered by TLR4. P, phosphorylation; R and C, regulatory and catalytic subunits of PKA.

To identify where in the signaling cascade cAMP was exerting its effects, we compared IL-6 message levels in control and AC-3 KD BECs. The absence of IL-6 message in AC-3 KD BECs after 1 h of exposure to bacteria suggested that cAMP was regulating IL-6 production at the transcriptional level rather than at the levels of translation or cytokine secretion. Since translocation of NF-κB into the nucleus of BECs following exposure to E. coli was largely unaffected in the AC-3 KD BECs (Figure 5B), cAMP may not be exerting its effect through altering NF-κB. Interestingly, one way that cAMP can directly promote expression of certain genes is to activate PKA, which translocates to the nucleus, where it phophorylates the transcriptional factor CREB [36]. Upon phosphorylation, CREB is believed to promote transcription of a number of genes, including IL-6, IL-8, IL-1α, and IL-1β, which possess consensus CRE sites on their promoter region [36,37,51–53]. Interestingly, in BECs, only IL-6 and IL-8 appear to be regulated by the cAMP/CREB pathway (Figure 5). That cAMP was modulating phosphorylation of CREB was evident from CREB phosphorylation in control BECs following exposure to E. coli but not in AC-3 KD BECs. Thus, taken together, our cumulative data reveal the existence of a distinct TLR4-activated signaling pathway in BECs involving Ca^2+^, cAMP, and phosphorylated CREB. A diagrammatic representation of the proposed TLR4-initiated Ca^2+^-, cAMP- and CREB-dependent pathway, as well as the NF-κB pathway in BECs, is shown in Figure 8. Although in the figure we have indicated that the cAMP/CREB pathway in BECs is activated by TLR4, our data also suggest that TLR2 and TLR3 activation may also trigger this pathway (Figure 6E).

Our analysis of the kinetics of IL-6 secretion in WT and AC-3 KD BECs has revealed that this novel second messenger/CREB-mediated pathway is mediating a faster IL-6 response than the classical NF-κB–mediated pathway. Following exposure of BECs to E. coli ORN103(pSH2), marked phosphorylation of CREB was observed at least 1 h before nuclear translocation of NF-κB was evident. Indeed, the earliest evidence of nuclear translocation of NF-κB in BECs following exposure to E. coli was at 2 h (Figure 5B). Another piece of evidence implicating the second messenger/CREB pathway in a rapid and distinct IL-6 response was the observation that a message for IL-6 was detectable in control BECs 1 h following exposure to *E. coli,* whereas no message for IL-6 was seen in AC-3 KD BECs. However, by 6 h after the classical NF-κB signaling pathways had been activated, there was very little difference in the amounts of mRNA in both cell types (Figure 5D). Consistent with this finding, the kinetics of IL-6 secretion by WT BECs and AC-3 KD BECs following exposure to E. coli ORN103(pSH2) revealed a 3-h lag in the latter's response, but by 12 h the amounts of IL-6 secreted were comparable (Figure 6A). Thus, the rapid and vigorous inflammatory responses to infection typically observed in the urinary tract may be attributable, at least in part, to this distinct cAMP-dependent signaling pathway. The relevance of the early IL-6 response by BECs may be linked to their role as first responders. One of the primary cell types in the urinary tract reacting to BEC-generated IL-6 are also BECs. These cells possess IL-6 receptors [54] and this cytokine, acting in autocrine fashion, may trigger various antimicrobial responses, such as production of antimicrobial peptides [55] and mucins, [56] as well as promote exfoliation of infected BECs.

The existence of multiple pathways in BECs for triggering IL-6 responses could be an adaptation to avoid inactivation by UPEC. Several recent studies have suggested that host-adapted pathogens possess the intrinsic capacity to block NF-κB activation in macrophages and cultured human epithelial cells through release of toxins or proteases [13,57–60]. Hunstad et al. have recently identified several genes *(rfa, rfb,* and *surA)* in UPEC that contribute to suppressing the cytokine responses of BECs [14]. Our observations that primary BECs (Figure 7) evoked a more modest IL-6 response to clinical UPEC CI5 compared to the laboratory E. coli strain and that the cAMP/CREB–mediated IL-6 response to UPEC CI5 in BEC lines was not striking compared to E. coli ORN103(pSH2) (Figure 6A and 6B) could be manifestations of this phenomenon. Conceivably, depending on the nature of the BECs, the UPEC CI5 strain is able to partially diminish one or both of the two TLR4-mediated signaling pathways leading to IL-6 secretion.

Finally, because of the rapid emergence of multi-resistance among UPEC isolates, there is mounting interest in the development of alternate antimicrobial strategies. One approach is to bolster innate immune defenses in the urinary tract either before or during infection. In this regard, our finding that small molecule enhancers of intracellular levels of Ca^2+^ and cAMP are sufficient to trigger early and vigorous cytokine responses from BECs is of interest. There are available many compounds capable of modulating the intracellular levels of both Ca^2+^ and cAMP [31,61–63]. Judicious application of some of these agents for the treatment and prevention of urinary tract infections is a possibility that will require further examination.

## Supporting Information

Table S1 Gene-Specific Primers Used in This Study (16 KB PDF)

Text S1 KD Creation and Western Blot Analysis Methods (62 KB PDF)

## Abbreviations

AC: adenylyl cyclase
BEC: bladder epithelial cell
[Ca^2+^]_i_: intracellular Ca^2+^
cAMP: cyclic adenosine monophosphate
CREB: cAMP response element–binding protein
GAPDH: glyseraldehyde-3-phosphate dehydrogenase
HEK: human embryonic kidney
KD: knockdown
LPS: lipopolysaccharide
PKA: protein kinase A
PMB: polymyxin B
PRR: pattern recognition receptor
RNAi: RNA interference
TLR: Toll-like receptor
UPEC: uropathogenic Escherichia coli
