# Supplementary material for: A Novel TLR4-Mediated Signaling Pathway Leading to IL-6 Responses in Human Bladder Epithelial Cells
Source: PLoS Pathog. 2007 Apr 27;3(4):e60. doi: 10.1371/journal.ppat.0030060 (PMC1857715; doi:10.1371/journal.ppat.0030060)
Supplement: Table S1 — (16 KB PDF) [file ppat.0030060.st001.pdf]

Table S1. Gene-specific primers used in this study.

	Sense	Antisense	Product size
AC-1	5'-CTGAGAGGATTTTCCACAAG-3’	5'-TTTGTGATATGAACCTTCCC-3’	466 bp
AC-2	5'-CTTCCTCTTCATCATCTTCG-3’	5'-TTCATTCTCCTTTGCAATTT-3’	574 bp
AC-3	5'-TACTCGGTGGAGAAGGAGAAGCAG-3’	5'-CGAAAACGCTTGTGGTCGTATTC-3’	554 bp
AC-4	5'-GACTTCAACCCACTGACACT-3’	5'-AGTCTGATGATGTTGGGAAG-3’	397 bp
AC-5	5'-GTACTCCAAGC AGGTAGACG-3’	5'-GCTGTAGGTGAAGTACTCGG-3’	484 bp
AC-6	5'-CATCTGCACACACTATCCAG-3’	5'-ATGGCCTCAATCATGTCTAC-3’	465 bp
AC-7	5'-CTCTCTGTGCTGATGTACGTCGAG-3’	5'-TCTTGACGTAGAGGCTGTGGAAGT-3’	587 bp
AC-8	5'-TTAGGAACCCCTCCTCCG-3’	5'- TGCTAGGGGCACAGTCAAG-3’	476 bp
AC-9	5'-GAAAACGGACGCCCACTTTG-3’	5'-TCC TCCAGGAAGAACACCATCC-3’	375 bp
soluble AC	5'-AAAACCTGTCACCTCC AACG-3’	5'-TCTAAAGCGTTGAG CCGAAT-3’	556 bp
GAPDH	5’-ATCCCATCACCATCTTCCAG-3’	5’-CCTGCTTCACCACCTTCTTG-3’	579 bp
IL-6	5'-ATGAACTCCTTCTCCACAAGCGC-3'	5'-GAAGAGCCCTCAGGCTGGACTG-3'	628 bp
IL-8	5'-GGACAAGAGCCAGGAAGAAACCACC-3'	5'-GCAACCCTACAACAGACCCACAC-3'	460 bp
IL-1a	5'-GTAAGCTATGGCCCACTCCA-3'	5'-AGCAGCCGTGAGGTACTGAT-3'	354 bp
IL-1b	5'-GGACAAGCTGAGGAAGATGC-3'	5'-TCTTTCAACACGCAGGACAG-3'	360 bp
