# Supplementary material for: A Novel TLR4-Mediated Signaling Pathway Leading to IL-6 Responses in Human Bladder Epithelial Cells
Source: PLoS Pathog. 2007 Apr 27;3(4):e60. doi: 10.1371/journal.ppat.0030060 (PMC1857715; doi:10.1371/journal.ppat.0030060)
Supplement: Text S1 — (62 KB PDF) [file ppat.0030060.sd001.pdf]

Text S1

Creation of Knockdowns Using RNA Interference

RNA interference vectors were generated using pQCXIN retroviral vector (BD Biosciences). Briefly, pQCXIN was digested by *Bam*HI and *Eco*RI and then was religated to generate pQCXIN1. Human U6 small nuclear RNA promoter was PCR-amplified from pTZ U6 + 1 (gift from John Rossi, Beckman Research Institute of the City of Hope, Duarte, CA) with added *Bgl*II site (5' ends), *Bam*HI, and *Xba*I sites (3' ends). The PCR product was cloned to the *Bgl*II and *Xba*I sites of pQCXIN1 to generate pQCXIN-U6. The following oligonucleotides were ordered from Integrated DNA Technologies, Inc.: AC3a, 5'-GATCC**GCTGTCTCCAGTACT ACAC**TTCAAGAGAGTGTAGTACTGGAGACAGCTTTTTTT-3’, and AC3b, 5'-CTAGA AAAAAA**GCTGTCT CCAGTACTACAC**TCTCTTGAAGTGTAGTACTGGAGACAGCG-3’; AC4a, 5’-GATCCG**TGTGTCCTCCATGATTTCC**TTCAAGAGAGGAAATCATGG AGGACACACTTTTTTT-3’, and AC4b, 5'-CTAGAAAAAAAG**TGTGTCCTCCATGATTT CC**TCTCTTGAAGGAAATCATGGAGGACACACG-3'; AC6a, 5’-GATCCG**TGACAGG TGTGAATGTGAA**TTCAAGAGATTCACATTCACACCTGTCACTTTTTTT-3’, and AC6b, 5'-CTAG AAAAAAAG**TGACAGGTGTGAATGTGAA**TCTCTTGAATTCACATTCA CACCTGTCACG-3'; AC7a, 5’-GATCCG**CTGGTGCTCGGTTCT TTGA**TTCAA GAGATCAAAGAACCGAGCACCAGCTTTTTTT-3’, and AC7b, 5'-CTAGAAAAAA AG**CTGGTGCTCGGTTCTTTGA**TCTCTTGAATCAAAGAACCGAGCACCAGCG-3'. The boldface

and underlined sequences are forward and reverse sequences, respectively, which correspond to nucleotides 2324–2342 of the human AC-3 gene (AC3a and AC3b, GenBank™ accession number AF033861), nucleotides 2096-2114 of the human AC-4 gene (AC4a and AC4b, GenBank™ accession number AF497516), nucleotides 2085-2103 of the human AC-6 gene (AC6a and AC6b, GenBank™ accession number AF250226), and nucleotides 747-765 of the human AC-7 gene (AC7a and AC7b, GenBank™ accession number NM_001114). The oligos were annealed to form double-stranded DNA and cloned into the *Bam*HI and *Xba*I sites of pQCXIN-U6 to generate pSi-AC3, pSi-AC4, pSi-AC6, and pSi-AC7. The Amphopack-293 Cell Line (BD Biosciences) was used to produce the viral particles. Production of viral particles, infection of target cell line (5637), and selection of viral infected cells were performed as recommended by the vendor of the pQCXIN vector (BD Biosciences). The geneticin-resistant stable-transfected cell lines were named AC-3 KD, AC-4 KD, AC-6 KD, and AC-7 KD. Knockdowns were verified by RT-PCR using the AC isoform-specific primers listed above.

In order to create TLR4 knockdown, the following oligonucleotides were synthesized: TLR4a, 5’-GATCCG**TTCCGATTAGCATACTTAG**TTCAAGAGACTAAGTATGCTAATCGGAACTTTTTTT-3’, and TLR4b, 5'-CTAGAAAAAAAG**TTCCGATTAGCATACTTAG**TCTCTTGAACTAAGTATGCTAATCGGAACG-3'. The boldface and underlined sequences are forward and reverse sequences, respectively, which correspond to nucleotides 1026-1044 of the human TLR4 gene (GenBank™ accession number U88880). Knockdowns were verified by RT-PCR using the gene-specific primers. The primer sequences for the

gene were as follows: 5'-CGATTCCATTGCTTCTTG-3' (sense) and 5'-GCTCAGGTCCAGGTTCTT-3' (antisense) for TLR4.

Western blot analysis

BECs were seeded onto 60-mm culture dishes and grown overnight. The cells were uninfected or infected with *E. coli* (MOI=100) for 1 h, or treated with 100 μg/ml LPS for 6 h. Cells were lysed in a RIPA buffer (Bio-Rad) containing 1 mM PMSF and a 1:100 dilution of mammalian protease inhibitor cocktail (Sigma). The cell suspension was passed 20 times through a 21-gauge needle and then sonicated. The cell lysate was centrifuged at 10,000 rpm for 10 min with the precipitates then being discarded. Protein concentrations were determined using the Bradford reagent (Bio-Rad) with bovine serum albumin as the standard. Cellular proteins (100 μg per lane) were electrophoresed in 4-15% sodium dodecyl sulfate-polyacrylamide gel electrophoresis (SDS-PAGE). Proteins were transferred to a PVDF (polyvinylidene fluoride) membrane, which was blocked with 5% non-fat dried milk in TBST (20 mM Tris-HCl, 150 mM NaCl, and 0.1% Tween 20, pH 7.5) for 1h, and then incubated overnight with 1:250-1:500 diluted anti-AC3 antibody (FabGennix, Inc.) in blocking solution at 4°C. After washing with TBST, the membrane was incubated with horseradish peroxidase-conjugated anti-rabbit IgG antibody for 1h at room temperature. The blots were then immunodetected with the enhanced chemiluminescence detection system (Pierce). As a loading control, 20 μg cellular proteins were loaded in 4-15% SDS-PAGE and blotted with anti-β-actin antibody (1:5000 dilution; Sigma). Image J software (National Institutes of

Health) was used for densitometry to quantify protein expression for statistical analysis.

Anti-CREB and anti-phospho-CREB antibody were purchased from Cell Signaling technology and CREB western blotting was performed according to the manufacturer's instruction. BECs were cultured onto 6-well plates overnight and left uninfected or infected with 100 MOI *E. coli* for 1 hr, or treated with 50 μM forskolin or 10 μM calcium ionophore A23187 for 1 hr. When specified, BECs were treated for 6 hrs with either 2 μg/ml of Lipoteichoic acid (TLR2 ligand) or 25 μg/ml of polyinosine-polycytidylic acid (TLR3 ligand).
